# Time measurement in insect photoperiodism: external and internal coincidence

**DOI:** 10.1007/s00359-023-01648-4

**Published:** 2023-09-12

**Authors:** David S. Saunders

**Affiliations:** The University of Edinburgh (Professor Emeritus), Edinburgh, UK

**Keywords:** Photoperiodism, Time measurement, External and internal coincidence, *Sarcophaga*, *Nasonia*

## Abstract

The identity and nature of the photoperiodic photoreceptors are now quite well known, as is the nature of the endocrine regulation of the resulting diapauses. The central problem of time measurement—how the photoperiodic clock differentiates long from short days—however, is still obscure, known only from whole-animal experiments and abstract models, although it is clearly a function of the insect circadian system. This review describes some of these experiments in terms of oscillator entrainment and two widely applicable photoperiodic clock models, external and internal coincidence, mainly using data from experiments on flesh flies (*Sarcophaga* spp) and the parasitic wasp, *Nasonia vitripennis*.

## Introduction

Most insect species that inhabit northern latitudes pass the winter in a state of dormancy (or diapause) controlled by hormones that are regulated by shortening day length (or increasing night length) as the seasons change (Denlinger 2022). In recent years, considerable advances have been made in determining the genetic basis of this phenomenon, with demonstrations that circadian ‘clock’ genes are often causally involved (Goto 2022). However, many studies of this sort are restricted to a comparison between a *single* long-day cycle (e.g., LD 16:8) and a *single* short-day cycle (e.g., LD 12:12) taken to represent typical summer and autumnal photoperiods, respectively (e.g., Kotwica-Rolinska et al. 2017; Benetta et al. 2019; Kontogiannatos et al. 2017; Barbera et al. 2017).

Whilst such experiments may indicate that ‘clock’ genes are involved in insect photoperiodism, they leave a more detailed analysis of this phenomenon incomplete, particularly the nature of the time measurement involved. To address this problem, the wealth of evidence from more traditional, non-invasive or ‘whole-animal’ experiments must also be considered. Such experiments include: (1) subjecting insects to the full range of photophases from the very short to the very long (and also to continuous darkness [DD] and continuous light [LL]) to provide a ‘complete’ photoperiodic response curve (PPRC); (2) the use of two (or more) short light pulses per cycle referred to as ‘symmetrical skeleton’ photoperiods; (3) ‘night interruption’ experiments or asymmetrical ‘skeletons’ in which the scotophase is systematically interrupted by a scanning light pulse; or (4) combinations of these approaches in light–dark cycles whose periods differ from 24 h. In addition, the roles of temperature and of light intensity must also be included, together with the nature of ‘downstream’ processes such as the photoperiodic ‘counter’ mechanism accumulating the effects of successive light cycles during the photoperiodic sensitive period (Saunders 1981a, b). These experiments are based on canonical properties of the insect circadian system and where ‘positive’ in their outcome offer persuasive evidence in favour of Bünning’s general hypothesis (Bünning 1936, 1960) that photoperiodic timing also has a circadian basis. Experiments describing such ‘whole animal’ photoperiodic responses and their interpretation are scattered over 7 decades or more (see Pittendrigh and Minis 1964; Pittendrigh 1966; Saunders 2002). The present review now combines the results of these earlier data with the more recent molecular observations in an attempt to provide a more complete description of the photoperiodic mechanism. Particular importance is placed on the results of skeleton photoperiods and night interruption experiments and on differences between two models for the photoperiodic clock—external and internal coincidence—first addressed by the author in the 1970s (Saunders 1975b).

## Quotation from Pittendrigh and Minis (1964) American Naturalist 98: 261–294

*Bünning’s general proposition, that circadian rhythmicity underlies the photoperiodic time measurement, is, in our view, correct. In the first place, the proposition seems highly plausible, *a priori*, in view of the diversity of other chronometric functions such as rhythms’ subserve. In the second place, there is a large body of experimental fact that cannot reasonably be interpreted in any other way. A “Coincidence Model” for photoperiodic induction is outlined; it is essentially Bünning’s original scheme given in somewhat more explicit terms. It may yet prove true that the “coincidence-device” type of model will prove inadequate; but that would not necessarily render Bünning’s more general proposition invalid that the circadian system somehow executes the time measurement. In any event, we note that any general theory of circadian oscillations as photoperiodic clocks must go well beyond the terms in which the proposition was first stated; and specifically, it must incorporate a general theory of the entrainment of such oscillations by light. Classical photoperiodic induction is, in our view, only an extreme case of the general seasonal modulation of the state of the circadian system that photoperiod (as the entraining agent) effects. The theory of entrainment outlined here gives an explicit basis for interpreting the action of night interruptions; and, among other things, indicates that the concept of “scotophil” needs more rigorous definition. As a specific fraction of the circadian cycle, it must be defined in terms of SCT* [Subjective Circadian Time] *time. Its discussion in terms of AZT time* [Arbitrary Zeitgeber Time] *(which is common in the literature) leads to a confounding of two distinct functions of the light: entrainment and induction. The most direct and unequivocal test of any theory involving the coincidence of a specific fraction of the circadian cycle with light is offered by the theory of entrainment for short pulses. Entrainment to T values close to τ involves fully predictable coincidence, or non-coincidence, of the short light pulse with particular SCT phases. The one such test so far performed supports the coincidence model. The possibility of two distinct pigments underlying the two distinct functions of light is raised.*

## Overt behavioural rhythms in insects

Four rhythmic systems form the basis of the present study. In the first, the rhythm of locomotor activity of the diurnally active adult blow fly *Calliphora vicina* (Kenny and Saunders 1991; Saunders 2021) shows the characteristics of rhythmicity in a single insect; this species also passes the winter in a state of larval diapause (Vinogradova and Zinovjeva 1972; Saunders 1987). The endogenous, circadian nature of the adult fly’s rhythm is revealed upon its transfer from a light–dark cycle into constant darkness (DD), whereupon the periodicity of activity persists (free-runs) with a period τ h differing somewhat from an exact 24 h (Kenny and Saunders 1991). In DD free-run, the oscillation passes through successive phases of subjective day (during which time most activity of this diurnal insect occurs) followed by phases of subjective night, when little activity is evident.

Short light pulses falling during the subjective day have little or no effect on the rhythm, but pulses falling in the early subjective night induce increasingly great phase delays (−Δφ *h*), whereas those falling in the late subjective night produce phase advances (+ Δφ *h*) to give a phase response curve (PRC) for that particular pulse ‘strength’. Entrainment of the activity rhythm to light cycles may then be compared with diapause induction, using overt rhythmicity as visible ‘hands’ of the otherwise covert system controlling diapause induction itself (see Kenny and Saunders 1991; Saunders 2021).

The second rhythmic system considered here is that of pupal eclosion (adult emergence) of *Drosophila pseudoobscura,* based on the now classical studies by Colin Pittendrigh and his co-workers, first at Princeton in the 1950s and 1960s, then to Stanford from 1969, and finally to the Hopkins Marine Biological station in Monterey, California, from 1971 until his retirement. This rhythm differs from that in the blow fly in that pupal eclosion is a ‘once-in-a-lifetime’ event and consequently is only evident in a mixed-age population. Nevertheless, Pittendrigh’s extensive data showed that a circadian oscillation persisted throughout each fly’s development, eclosion occurring in a narrow ‘gate’ close to dawn. As with rhythms in individual insects*,* pulses of light falling in the early subjective night caused increasingly large phase delays, whereas those falling in the late subjective night caused phase advances to give a phase response curve (PRC) that was considered to represent the rhythm’s ‘time course’. Although *D. pseudoobscura* was non-photoperiodic and without a diapausing stage, Pittendrigh (Pittendrigh and Minis 1964) was quick to realise that its circadian rhythm PRCs within a framework of entrainment could be used to address Bünning’s hypothesis (1936, 1960) that circadian rhythmicity provides the ‘clockwork’ for photoperiodic time measurement.

**Fig. 1:**
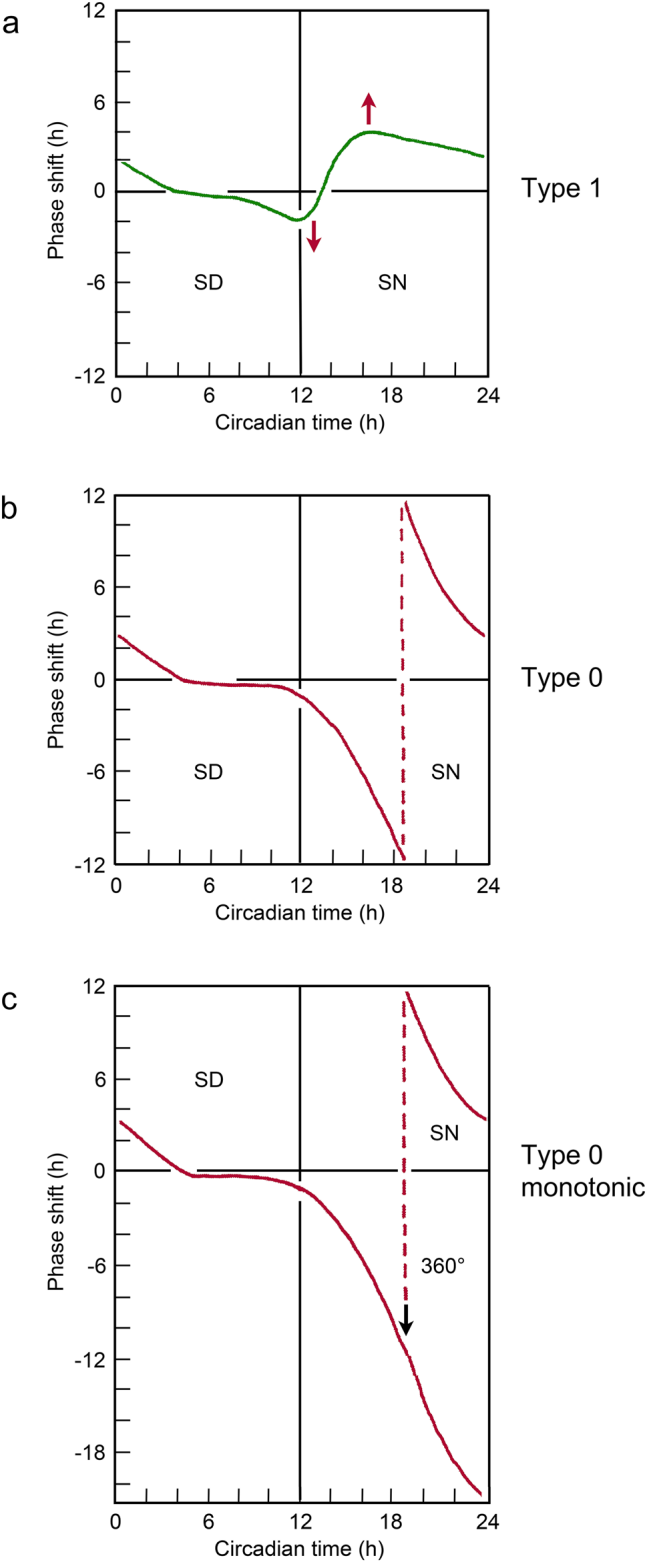
Phase response curves (PRCs) and entrainment (schematic). Each panel shows one cycle of a free-running circadian rhythm with two half-cycles, subjective day (SD) and subjective night (SN). **a** The PRC (green curve) is of the low ‘amplitude’ type 1 with small delaying phase shifts (= Δφ h) in the late SD and early SN and advancing phase shifts (+ Δφ h) in the late SN produced by systematic probing with a short supplementary pulse of light. After type 1 PRCs subsequent activity maxima occur roughly parallel to the *beginning* of the scanning pulse. Increasingly ‘strong’ pulses (longer duration and/or greater illuminance) induce larger phase shifts (red arrows). **b** With increasing pulse ‘strength’, the PRC (red curve) is of the high ‘amplitude’ type 0 with phase shifts reaching the maximum at + 12 and −12 h separated by a data-less section. **c** Removal of the data-less section from the type 0 PRC produces a monotonic curve that treats all phase shifts as delays. Further increases in pulse strength (longer duration and/or increased illuminance) result in a straight-line response parallel to the *end* of the light pulse (see Fig. 2) a phase equivalent to the beginning of the subjective night (Pittendrigh 1960). Redrawn from various sources (Pittendrigh 1960; Pittendrigh et al. 1991; Winfree 1970)

Pittendrigh’s early work on *D. pseudoobscura* showed that magnitude of the phase shift was a result of the ‘strength’ of the pulse, with reciprocity between pulse duration and light ‘intensity’, short pulses of higher light intensity being equivalent to longer pulses of lower intensity (Pittendrigh 1960). Winfree (1970) later coined the terms Type 1 for PRCs produced by very short or low intensity light pulses and Type 0 for those produced by the stronger pulses. With Type 1 responses, the phase changes are relatively small, resulting in response curves that are still roughly ‘parallel’ to the onset of the light pulse, whereas in Type 0, the phase changes are larger, eventually resulting in curves that are approximately parallel to light-off (Fig. 1). An observation of particular importance in that context was that the oscillation governing eclosion was reset to a phase equivalent to the beginning of the subjective night (Circadian time, CT 12 h) after a photophase of 12 h or more, or after transfer from constant light (LL) into darkness (Pittendrigh 1966). This important property of the system is also shown in *C. vicina* where the circadian oscillation is set to a phase equivalent to CT 12 h after a lengthy exposure to light, regardless of the phase of the oscillation at light-on (Saunders 2021).

The third species discussed here is the flesh-fly *Sarcophaga argyrostoma* which, like *D. pseudoobscura,* shows a population circadian rhythm controlling adult emergence, but—unlike the latter—also enters a robust diapause in the pupal instar (Saunders 1971). It therefore offers the possibility of testing Bünning’s hypothesis (1936, 1960) in a species presenting both diapause and an easily monitored rhythm. However, whereas entrainment of the eclosion rhythm in *D. pseudoobscura* is sensitive to light pulses throughout larval and pupal development, both diapause induction and entrainment of circadian rhythms of eclosion in the flesh-fly come to an end by the time the fully fed larva leaves its food and burrows into the soil to pupariate.

The fourth species considered in this review is the parasitic wasp *Nasonia vitripennis,* a pupal parasite of *S. argyrostoma* and a wide range of other flies, including *C. vicina.* The photoperiodic clocks in *Sarcophaga* and *Nasonia* are of particular interest here, because they resemble two principal models: external and internal coincidence, respectively (Saunders 1978).

## Circadian entrainment and phase response curves in the analysis of photoperiodic timing

Although *D. pseudoobscura* was found to be day-neutral and without a diapause in its life cycle, Pittendrigh soon concluded that the time measurement inherent in photoperiodic induction could be explained by comparison with an insect’s circadian responses (see, for example, Pittendrigh and Minis 1964; Pittendrigh 1966). The following sections describe subsequent tests of that notion in *S. argyrostoma*.

### The ‘complete’ photoperiodic response curve (PPRC)

‘Complete’ PPRCs for insect diapause induction have been described on numerous occasions (Lees 1955; Saunders 2001; Denlinger 2022); these cover the full range of photophases, natural and unnatural, together with continuous darkness (DD) and continuous light (LL). Responses to light–dark cycles outside the natural range for the latitude of origin are included, since any clock model proposed must also explain such data. Ultra-short photoperiods, for example, may be defined as those not occurring naturally at the insect’s latitude or during the winter when the insect is already in diapause and therefore unresponsive. The proportion entering diapause in these ultra-short photoperiods is variable and generally reduced in comparison with that in ‘strong’ natural short days.

**Fig. 2:**
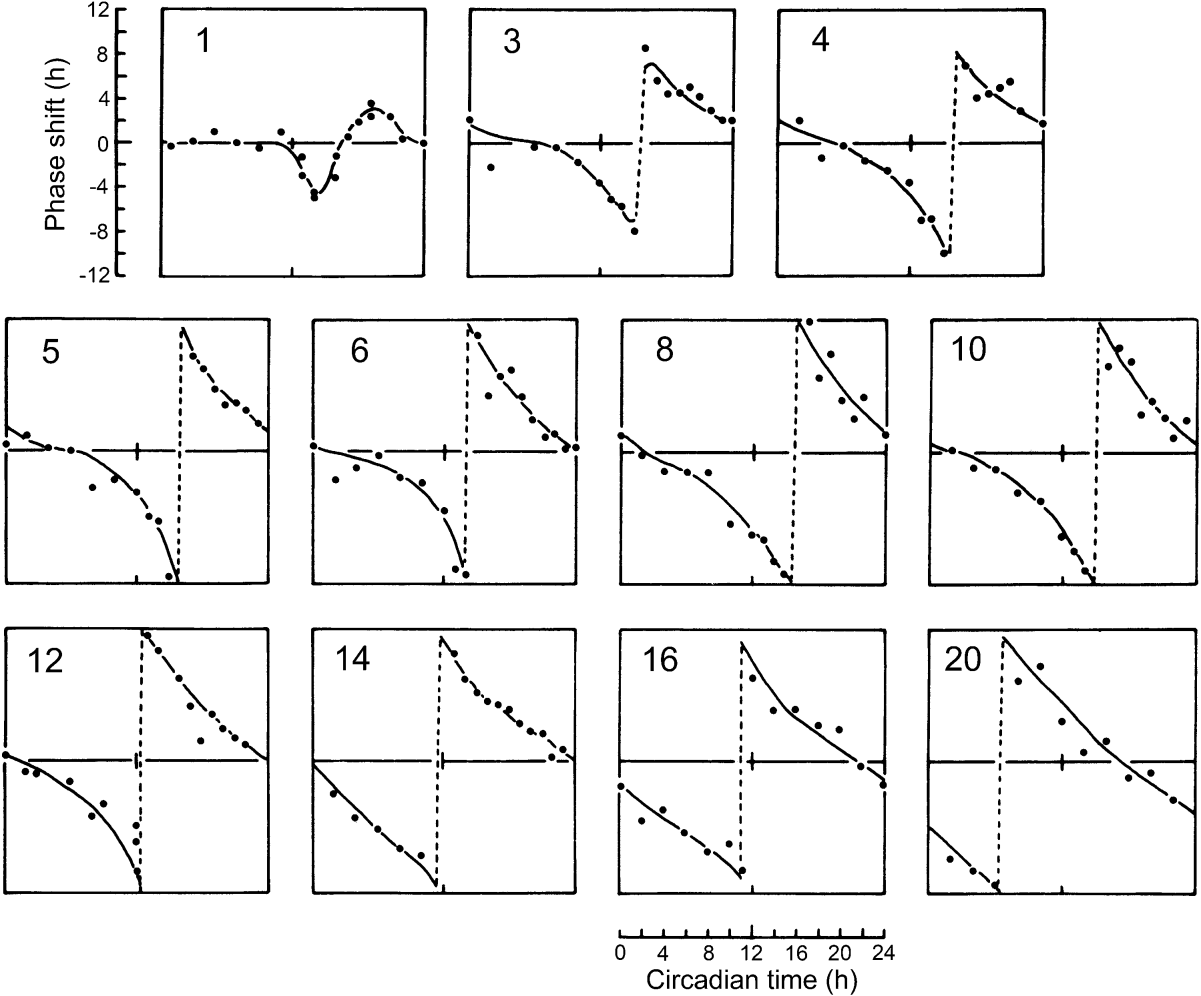
Phase response curves (PRCs) for the adult emergence (pupal eclosion) rhythm of the flesh-fly *Sarcophaga argyrostoma* exposed as larvae to light pulses of 1–20 h. Short pulses (1–3 h) give rise to Type 1 PRCs in which the resulting curve is roughly parallel to the beginning of the pulse. Longer and ‘stronger’ pulses give rise to Type 0 PRCs with resulting curves roughly parallel to the end of the pulse. From Saunders (1981b)

The reason for this reduction in diapause in ultra-short photoperiods in the flesh fly was investigated by establishing PRCs for pulse durations ranging from 1 to 20 h (Fig. 2) (Saunders 1978) on first instar larvae, a stage when photoperiodic sensitivity was most marked. Pulse lengths of 1 to 3 h were found to give rise to low-amplitude type 1 responses, whereas those of longer pulses produced responses approaching or reaching type 0. Light pulses of 12 h or more reset the oscillation to near constant phase equivalent to the onset of the subjective night (CT 12 h) (Saunders 1978) as in *D. pseudoobscura* and *C. vicina.*

In the eclosion rhythm of *D. pseudoobscura* (Pittendrigh and Bruce 1957)—and probably also in *S. argyrostoma—*eclosion behaviour is regulated by a two-tier system comprising a light-sensitive pacemaker that, in turn, controls a more downstream and temperature-sensitive slave. In *D. pseudoobscura,* pacemaker phase shifts are achieved almost immediately in response to a light pulse (Chandrasekaran 1967) but observable transients occur, both to the pacemaker and to the slave oscillator ‘catching up’ with the former. In doing so, type 1 responses induce only small phase shifts and pass through a larger number of transient cycles before achieving steady state, whereas stronger pulses giving rise to type 0 responses induce larger phase shifts and more rapid entrainment. It is probable that diapause induction is regulated in the same way.

**Fig. 3:**
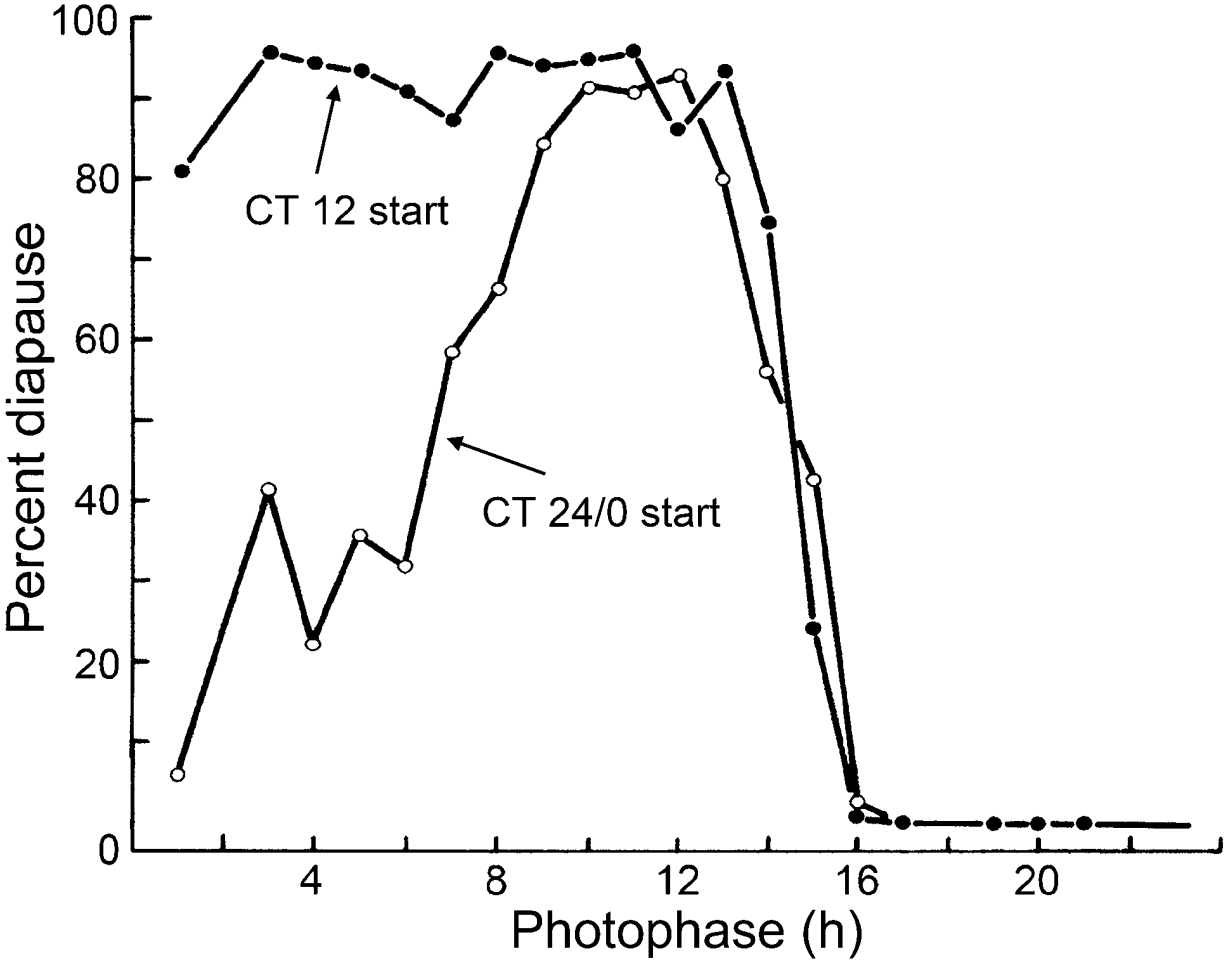
*Sarcophaga argyrostoma.* Photoperiodic response curves (PPRCs) for light pulses of 1–21 h in duration with the first pulse in the train starting either at CT 12 (close to final steady-state entrainment) or a full half-cycle away at CT 24/0 (far from entrainment). With shorter or ‘weaker’ light pulses (less than 8 h), pulses starting at CT 12 h induce levels of pupal diapause comparable to those engendered by longer, stronger pulses, whereas those starting at CT 24/0 h produce much lower diapause incidence. This suggests that diapause incidence depends on the rate of entrainment and, therefore, the number of transient cycles experienced before final steady state. Redrawn from Saunders (1982)

An experiment was therefore conducted (Saunders 1982, 2022) in which cultures of larvae were exposed to trains of light pulses (of 1–21 h in duration) with the light pulses starting already close to entrainment at CT 12 (dubbed in phase), or a full half-cycle away at CT 24/0 (dubbed out of phase) (Fig. 3). The number of transient cycles before steady state was estimated using a computer program based on the light pulse PRCs. Results showed that cultures of larvae initially out of phase and passing through a greater number of transients than cultures starting in-phase, resulted in a lower incidence of diapause. On the other hand, cultures starting in-phase produced levels of diapause equivalent to ‘strong’ short days of 8 h or more, suggesting that steady-state entrainment of the presumed photoperiodic oscillator facilitates full diapause induction.

**Fig. 4:**
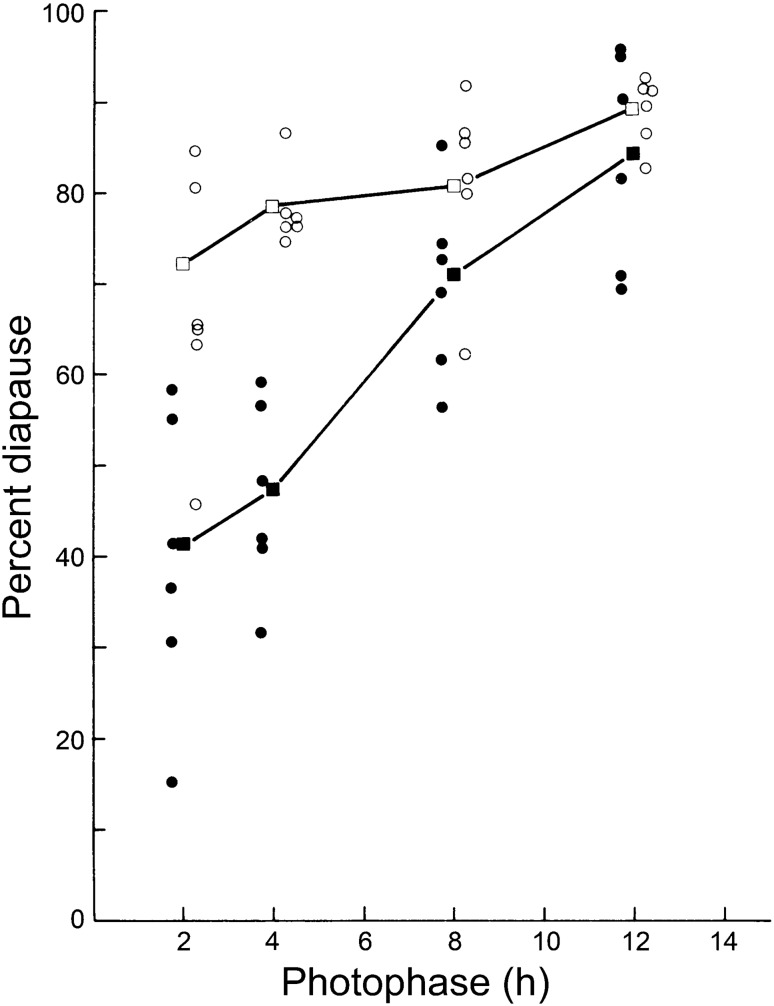
*Sarcophaga argyrostoma.* Incidence of pupal diapause produced by cultures of larvae exposed to ultra-short light pulses of 2, 4, and 8 h at light intensities of 240 µW cm^−2^ (closed circles) or to much higher irradiance of approximately 16,000 µW cm^−2^ (open circles). Increased irradiance induces a greater incidence of pupal diapause, probably because PRCs are raised from Type 1 to Type 0 and induce fewer transient cycles. Redrawn from Saunders (1982)

In a second experiment, light–dark cycles each containing 2, 4, 8, or 12 h of light, but with the irradiance of the photophase at either 240 μW cm^−2^ or much increased to about 16,000 μW cm^−2^ (Saunders 1982, 2022). Figure 4 shows that when the strength of the light pulse was increased, diapause incidence was also increased, particularly for the shorter photoperiods. This increase was not caused by an unavoidable rise in temperature at the increased irradiance, because higher ambient temperature in DD reduces diapause (Saunders 1971). Reciprocity between pulse duration and photophase intensity probably means that increased illuminance strengthened the ultra-short photoperiods from type 1 PRC towards that of type 0, thereby increasing diapause incidence by reducing the number of transients.

In *S. argyrostoma,* the phase shifting effects of 12 h light pulses were followed throughout larval development (Saunders 1979b) to delineate the photoperiodic sensitive period. Sensitivity began in the intra-uterine embryo then, in first instar larvae gave a type 0 PRC which subsequently declined through a series of type 1 responses until no response was recorded in third instar, post-feeding or ‘wandering’ larvae prior to their burrowing into the soil to pupate. Initially, this reduction in phase shifts during larval development was attributed to oscillator dampening. However, since the photoperiodic oscillator must continue through the pupal instar to time adult emergence, it seems more likely that the attenuation results from the photoreceptors becoming ‘detached’ from the oscillation. The effect, however, is the same: larval sensitivity to photoperiod steadily declines during larval development. In other species of flesh fly, this aspect shows variation. In North American *S. crassipalpis* (Denlinger 1971), for instance, sensitivity to photoperiod is largely restricted to the intra-uterine embryos, whereas in Japanese *S. similis,* it extends into the wandering larva itself (Goto and Numata 2009). Rapid attenuation of the photoperiodic response (likened to ‘oscillator dampening’) is also seen in some aphids—*Megoura viciae,* for example (Lees 1973)—and in some drosophilids at very high latitudes (Vaze and Helfrich-Fȍrster 2016; Kauranen et al. 2019; Tyukmaeva et al. 2020) in which the photoperiodic clock presents characteristics resembling an ‘hourglass’.

### Symmetrical ‘skeleton’ photoperiods and the bistability phenomenon

Pittendrigh and Minis (1964) showed that the eclosion rhythm of *D. pseudoobscura* could synchronise to a regime consisting of two short (15 min) pulses of light per cycle which they called a symmetrical ‘skeleton’ photoperiod (PPs) to distinguish it from a ‘complete’ photoperiod (PPc): this result attracted attention to the importance of the ‘on’ and ‘off’ transitions of the photophase as important signals in the entrainment phenomenon. Skeletons of short photophases up to about 11 h (PPs 11) were found to simulate almost exactly their complete counterparts (PPc 11), with the two 15 min pulses causing phase shifts in steady state that ‘balanced’ each other by the first causing phase delays (−Δφ) and the second, phase advances (+ Δφ) of the same magnitude but of opposite sign (Pittendrigh 1965). Attempts to entrain the eclosion rhythm to skeletons longer than about 14 h, however, were more complicated. A skeleton of PPs 14, for example, was open to two ‘interpretations’, one of about 14 h and the other of about 10 h. Between these conditions, the oscillator underwent a ‘phase jump’ and came to accept the shorter of the two steady states as ‘day’. Longer initial skeletons similarly jumped to the shorter alternative.

**Fig. 5:**
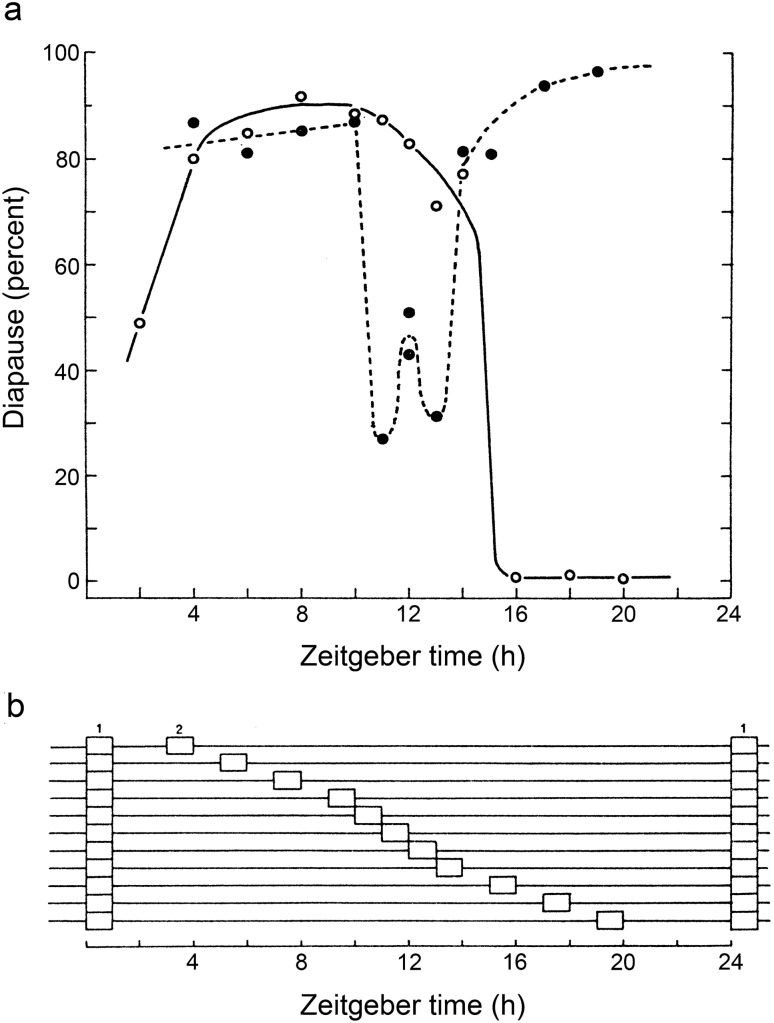
*Sarcophaga argyrostoma.* **a** Diapause incidence in cultures of larvae exposed to complete (PPc) photoperiods (open circles) or to symmetrical ‘skeleton’ (PPs) photoperiods formed from two 1-h pulses of light (closed circles). Complete photoperiods produce a response curve with a typical critical daylength. ‘Skeletons’ of PPs4 to PPs10 also produce a high incidence of pupal diapause similar to that produced by their complete counterparts. Attempts to entrain to skeletons longer than PPs14, however, lead to a phase jump to the shorter interpretation. This also induces a high incidence of diapause. Skeletons with an interval close to half the circadian period (11–13 h), produce less diapause. **b** Experimental design of the ‘skeleton photoperiods’. Redrawn from Saunders (1975a)

Similar experiments were conducted using *S. argyrostoma* with respect to the production of pupal diapause (Saunders 1975a) (Fig. 5). Symmetrical skeletons (PPs) of 4–10 h formed from two 1-h light pulses per cycle led to a high incidence of diapause similar to that of their complete (PPc) counterparts. Attempts to entrain to skeletons of 16 h or more, however, led to a phase jump to the shorter alternative, also inducing a high diapause incidence. Skeletons of 11, 12, and 13 h, close to half the circadian period, however, produced less diapause (27–51%) than their corresponding complete photoperiods. The behaviour of diapause induction in *S. argyrostoma,* was clearly regulated by the entrainment of an oscillatory system similar to that in *D. pseudoobscura.*

In *D. pseudoobscura*, symmetrical skeletons initially close to half the circadian period were found to present an additional property: the final steady state depended on two initial variables, (1) the *phase* illuminated by the first pulse in the train, and (2) the *value* of the first interval between the two pulses (Pittendrigh 1966). This could result in two alternative steady states called the ‘bistability phenomenon’, predicted by computer simulation and considered to be important evidence that pupal eclosion was indeed an oscillatory phenomenon.

Similar results were obtained for diapause induction in *S. argyrostoma* (Saunders 1975a) in which larval cultures were exposed to two skeleton regimes formed from 1-h light pulses, either LD 1:9:1 (PPs 11) or LD 1:13:1 (PPs15) each with the first light pulse in the train commencing at all circadian phases. These two skeletons were chosen, because the former, if taken as ‘day’, should produce a short-day, high incidence of diapause equivalent to that induced by an 11 h complete photophase, whereas the latter should act as a long day, producing a low incidence equivalent to that in a 15 h photophase. The results of this experiment (Saunders 1975a) produced two curves of diapause incidence, mirror images of high and low diapause, showing that the bistability phenomenon applied to diapause induction in a fashion equivalent to those of circadian entrainment in *D. pseudoobscura* described above. Similar ‘positive’ bistability results have since been observed in *C. vicina* (Vaz Nunes et al. 1990) and in the cabbage moth *Mamestra brassicae* (Kimura and Masaki 1993) and constitute strong evidence for the circadian basis of photoperiodic time measurement and diapause regulation.

### Asymmetrical ‘skeletons’ and night interruption experiments

**Fig. 6:**
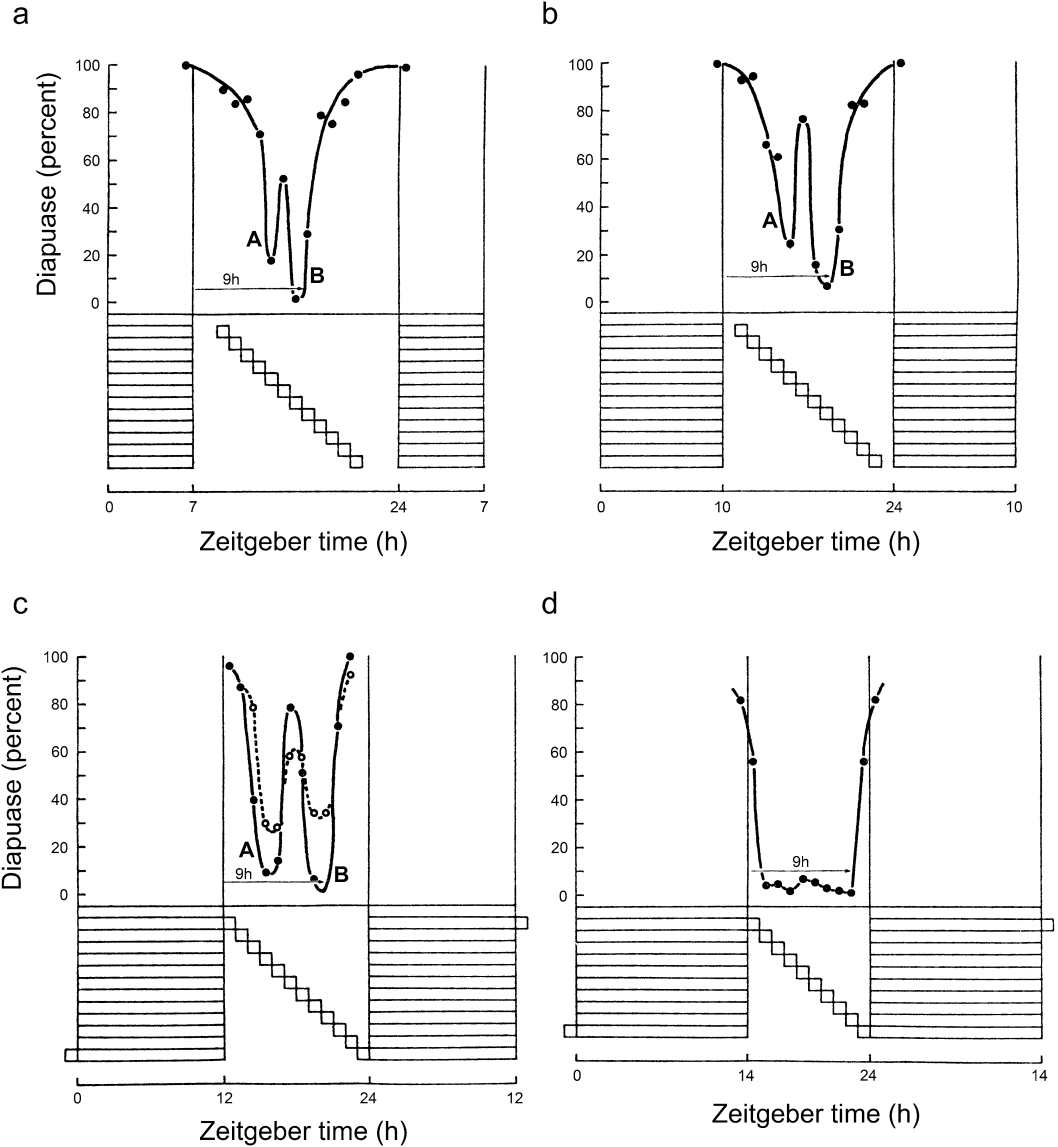
*Sarcophaga argyrostoma.* Diapause responses of larvae exposed to asymmetrical ‘skeletons’ (or night interruption experiments) using basic photoperiods of LD 7:17 to LD 14:10. In the three shorter photoperiods (panels **a**–**c**), scanning 1-h light pulses produced two phases of reduced diapause incidence (A and B), whereas with LD 14:10 diapause was reduced to near zero with all scanning pulses (panel **d**). In panels **a**–**c**, point B occurred about 9 h after the end of the main light component. This result suggests that point B is the diapause-regulating photoinducible phase φ_i_ (see text for details). Solid and dotted lines show data from replicate experiments. Redrawn from Saunders (1975a)

Interrupting the scotophase with additional short light pulses (‘night interruption’ experiments) were originally performed to investigate possible light-sensitivity phases during the dark period. Early results, often for the additional light pulse placed centrally in the night, seemed to indicate that such treatments were sometimes ineffective (Lees 1955). However, Adkisson (1964, 1966), working with the pink bollworm moth *Pectinophora gossypiella* and using systematic scanning of the scotophase by 1 or 2 h light pulses, showed that long-day effects (diapause avoidance) were observed at *two* phases, one early (point A) in the night and the other (point B) later (Fig. 6). Pittendrigh (1966) observed the similarity between these results and his own observations in *D. pseudoobscura*—which he called ‘asymmetrical skeletons’. Using PRCs, he showed that, in conjunction with the main photophase, the early supplementary pulse acted as a ‘new dusk’ and the later pulse as a ‘new dawn’ and constituted strong evidence for the circadian basis of insect photoperiodism. Such bimodal responses to night interruption experiments have since proved almost universal in the insects, including the flesh fly *S. argyrostoma* (Saunders 2002, 2020).

In *S. argyrostoma,* two further experiments were conducted using night interruption techniques but in non-24 h light–dark cycles (Saunders 1979a) inspired by earlier experiments with the green vetch aphid by Lees (1970). In the first test, flesh-fly larvae were exposed to a series of asymmetrical skeletons in which the supplementary pulse was placed early in the night (at point A) and the terminal hours of darkness systematically increased from 7 to 13 h. In the second test, the supplementary light pulse was placed later in the night at point B (9 h after light-off) and was then followed by 12 h of darkness, a period greater than the critical *night*length for diapause induction. Results of the first test showed that the diapause-averting effects of light falling on point A could be reversed by a terminal dark period longer than the critical *night* length, whereas results of the second test showed that the ‘long day’ diapause-averting effect of illuminating point B could *not* be reversed by the terminal diapause inducing long night. This latter result suggested that point B marked the position of the photoperiodic photoinducible phase. This evidence proved to be of crucial importance in understanding how the circadian system regulates diapause induction.

## Models for the photoperiodic clock in insects

Bünning’s original model for the photoperiodic clock was the first to suggest that light had two roles: in modern terminology, these are now recognised as phase setting (entrainment) and photoinduction. Studies of the pupal eclosion rhythm in *D. pseudoobscura* allowed Pittendrigh and Minis (Pittendrigh and Minis 1964; Pittendrigh 1966) to develop these ideas as their ‘Coincidence’ model (see quotation at the beginning of this review). Subsequently, however, Pittendrigh (1972) proposed several possibilities for a possible role for circadian rhythmicity, two of which were ‘external coincidence’ and ‘internal coincidence’. The former—equivalent to their original proposal—was seen as a *single* circadian oscillation with a *dual* role for light (entrainment and photoinduction), whereas the latter was seen to involve *two* (or perhaps more) oscillators, separately entrained by dawn and dusk, with a *single* role for light: that of entrainment. They observed that two distinct photoreceptors, one for entrainment and the other for photoinduction, were distinctly possible. Given that insect photoperiodism has probably evolved on numerous occasions, these models (and perhaps others) remain valid alternatives.

External coincidence is clearly appropriate for diapause regulation in both *S. argyrostoma* and *C. vicina* (Kenny and Saunders 1991; Saunders 2021). Three ‘components’ of the photoperiodic response are significant in this respect: (1) The first is Pittendrigh’s observation that the circadian oscillator is phase set after a protracted photophase to CT 12 h—the start of the subjective night. This suggests that the photoperiodic response at the critical photoperiod involves *night*length measurement. (2) The second is the observation that point B in night interruption experiments represents the photoperiodic sensitive point in the cycle (Pittendrigh’s ‘photoinducible phase’, or φ_*i*_). This ‘photoinducible phase’ occurs at the end of the critical *night* length (9–9.5 h)—therefore, φ_*i*_ is at CT 12 + 9.5 = CT 21.5 h. (3) The third is experimental evidence that point B at CT 21.5 h in *S. argyrostoma* is the phase of φ _i_ was afforded by the ‘Lees experiment’ (reviewed above) that showed that the diapause-averting effects of a light pulse falling in the late subjective night at about CT 21.5 h could not be reversed by a subsequent long night.

In natural populations of *S. argyrostoma,* larvae would be exposed to long days (short nights) at the end of summer, cycles in which φ_*i*_ would still be illuminated, leading to further nondiapause development. As the days shortened (or nights lengthened) with the approach of autumn, however, φ_*i*_ would start to fall in the dark, leading to pupal diapause. External coincidence operates in a similar fashion in the adults of *C. vicina* (Saunders 2021).

**Fig. 7:**
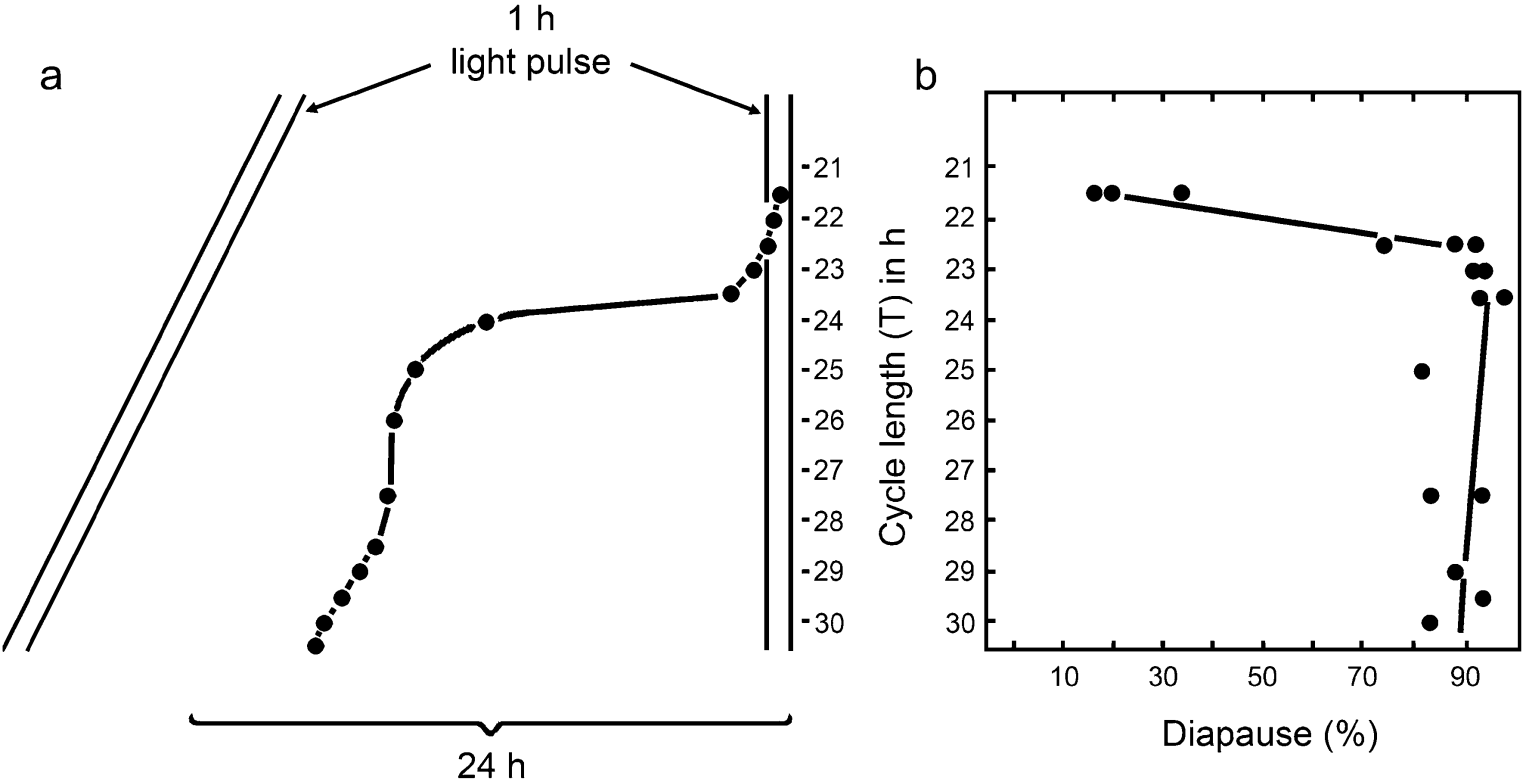
*Sarcophaga argyrostoma.* Pupal diapause induction in larval cultures exposed to single 1-h pulses of light in cycles (*T* h) across the range of primary entrainment of the circadian oscillator (τ). **a** Computed phase relationships of the photoinducible phase (φ_*i*_ at CT 21.5 h) to the light cycle (*T*). **b** Incidence of pupal diapause in these cultures. When *T* is less than the circadian period τ, the photoinducible phase φ_*i*_ coincides with the light pulse in each cycle and pupae develop without arrest, whereas when *T* is greater than τ, φ_*i*_ falls in the dark and diapause supervenes. Observed incidence of pupal diapause agrees with this prediction and confirms that the photoinducible phase lies at CT 21.5 h. Redrawn from Saunders (1979a, b)

Confirmation that φ_*i*_ in *S. argyrostoma* occurs at about CT 21.5 was obtained in a T-experiment (Saunders 1979a) in which groups of larvae were exposed to light–dark cycles, each containing a 1-h light pulse, but with the cycles ranging in period (*T* h) across the range of primary entrainment of the eclosion oscillator (Fig. 7). According to the properties of the circadian system outlined by Pittendrigh (Pittendrigh and Minis 1964; Pittendrigh 1965), when *T* is greater than the period of the oscillation (*T* > τ h), the light pulse must fall in the early subjective night (CT 12–18), whereas when *T* < τ *h*, it must fall in the *late* subjective night (CT 18–24). Simply by changing the value of *T*, therefore, the single 1-h light pulse per cycle could be made to illuminate different phases of the oscillator. Results (Fig. 7) showed that in all *T* cycles greater than 24 h, the presumed phase of φ_*i*_ (at CT 21.5 h) fell in the dark and diapause incidence was high, but when *T* was less than 24 h, φ_*i*_ was illuminated by the light pulse and diapause incidence was greatly reduced. The photoinducible phase, therefore, must be at point B.

**Fig. 8:**
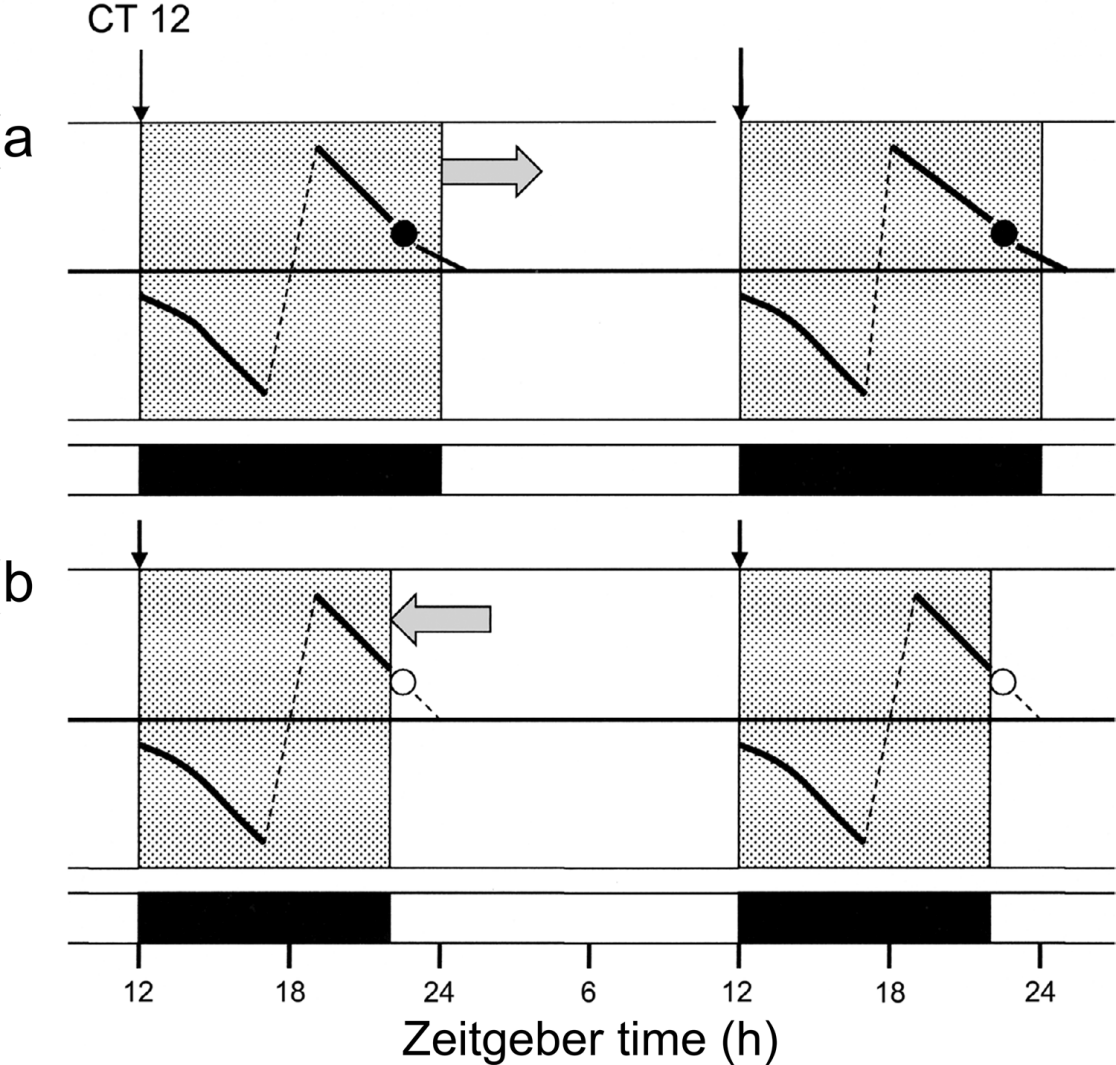
The external coincidence model of the photoperiodic clock in *Sarcophaga argyrostoma.* **a** In the long nights of autumn φ_*i*_ (closed circle ●) lies in the night and pupae enter diapause. **b** In the long days (or short nights) of summer (left pointing arrow) φ_*i*_ comes to lie in the light (open circle o) and the pupae develop to adult flies without arrest. Photoperiodic time measurement in the flesh fly is therefore regulated by dawn light coinciding, or not, with a light-sensitive phase (φ_*i*_) at about circadian time CT 21.5 h. A similar mechanism also occurs with larval diapause regulation in the blow fly *Calliphora vicina.* Redrawn from Saunders (2021)

Although the genetic basis of the *Sarcophaga* photoperiodic oscillator remains to be elucidated, further data concerning properties of φ_*i*_ are available. Working with Japanese populations of *S. similis,* Goto and Numata (2009) showed that point A in night interruption experiments was maximally sensitive to light at 470 nm or shorter, whereas that at φ_*i*_ (point B) showed a wider range from about 395 to 660 nm—a result like that produced by Lees (1981) for the aphid *Megoura viciae*. Identity of the photoreceptive molecules involved is currently unknown, but the blue-light sensitivity of both points A and B might involve cryptochrome (the circadian entrainment photoreceptor), whereas the longer wavelength sensitivity of φ_*i*_ at point B could be because the photoreceptor at this phase differs from that at A and is possibly opsin based. In a later study, Yamaguchi and Goto (2019) isolated four strains of *S similis* in Japan, from Hokkaido in the north (43.2 °N) to Kyushu in the south (32.75 °N) and showed that the position of φ_*i*_ varied with latitude, with a longer critical *night*length in the southernmost population. These data show that, of all the available clock models, external coincidence is most appropriate model for the photoperiodic clock in flesh flies (Fig. 8).

The photoperiodic clock in the parasitic wasp *Nasonia vitripennis* differs from that in the fly in several respects*.* In the wasp, maternal exposure to short days (long nights) leads to a larval diapause. Nanda–Hamner (NH) experiments (Nanda and Hamner 1958) in which adult females of the wasp were exposed to a range of light–dark cycles, each containing a fixed photophase coupled to a range of dark phases to provide overall cycle lengths up to 72 h or more, produced evidence for ‘dawn’ and ‘dusk’ oscillators, characteristic of internal coincidence (Saunders 1974). Additional evidence in favour of this model in *N. vitripennis* arose from the demonstration of a single role for light, i.e., entrainment of the oscillators, but not for diapause induction per se. Four pieces of evidence support this contention: (1) diapause induction could be regulated by temperature cycles in the absence of light (Saunders 1973), (2) induction of *non*diapause larvae continued in DD following a series of long-day cycles (Saunders 1978), (3) chilling in both photophase and scotophase effectively shortened both of those components (Saunders 1967), unlike in the flesh fly where only the scotophase is so affected (Saunders 1978), and (4) both early and late illumination of the scotophase with monochromatic light in *N. vitripennis* produced a similar action spectrum at both points (Saunders 1975b) with no evidence of a photoinducible phase at point B, as in the flesh fly (Goto and Numata 2009).

Strong evidence exists, therefore, to conclude that external coincidence is appropriate for the flesh fly, whilst internal coincidence is appropriate for the wasp. The following section attempts to relate these differences to current knowledge concerning the role of circadian clock genes in these phenomena.

## Circadian clock genes and insect photoperiodism

In *D. melanogaster,* there are five main ‘clock’ genes (plus a few more for fine-tuning) that regulate the autoregulatory feedback loops producing circadian rhythmicity; these are *period (per), timeless (tim), doubletime (dbt), clock (Clk),* and *cycle (cyc).* Their roles in the generation of circadian and photoperiodic responses have been reviewed recently by Goto (2022). In brief, after transcription of *per* and *tim*, their proteins, PER and TIM accumulate in the cytoplasm, reaching a maximum in late scotophase where they form a dimer (PER/TIM) that enters the nucleus to repress transcriptional activity of the CLK/CYC complex. The circadian cycle eventually starts again with the transcription of *per* and *tim*. The first part of this cycle is regarded as negative feedback, whereas activity of *Clk* and *cyc* as a positive element.

The role of clock genes in photoperiodic time measurement has attracted attention in recent years, sometimes producing conflicting results. Two main techniques have been used: (1) determination of clock gene mRNA titres in different photoperiods, generally under representative short and long daylengths, and (2) gene ‘silencing’ using the dsRNA technique aimed at determining the action of individual genes. Results of such experiments in a wide array of insects have been reviewed by Goto (2022) and will not be considered here in detail. However, comparable results for *Sarcophaga* spp and for *Nasonia vitripennis* will be considered, since these species have been taken to represent examples of external and internal coincidence, respectively (Saunders 1978).

Before these developments in the molecular biology of photoperiodism were investigated, Lewis and Saunders (1978) proposed a model for diapause regulation in insects—specifically for *S. argyrostoma—*based on Lewis’s earlier model for circadian rhythmicity in the weta *Hemideina thoracica* (Gander and Lewis 1979; Christensen and Lewis 1983). This was a control system feedback model that envisioned synthesis of a ‘substance c’ that rose to a peak in late scotophase, where an above-threshold value falling in the dark led to diapause induction but to nondiapause development if the peak coincided with light, as in external coincidence. The above-threshold value of ‘c’ was therefore seen as the embodiment of φ_*i*_.

Working with *S. crassipalpis*, Goto and Denlinger (2002) showed that peak expression of *per* mRNA occurred during the scotophase but was severely reduced during long days. Koštál et al. (2009) later showed that peak expression of *per* mRNA occurred during the scotophase during short days. It is tempting, therefore, to suggest that ‘substance c’ of Lewis’s model represents the protein PER or the PER/TIM dimer that accumulates in the cytoplasm during the scotophase to reach an above-threshold peak late in the night—acting as the photoinducible phase φ_*i*_. ‘Substance c’ is thus a product of translation rather than transcription. Goto and Numata (2009) (reviewed above) have also shown that action spectra for light impinging on φ_*i*_ suggest the involvement of two photoreceptors, one possibly CRYd absorbing in blue light for entrainment and the other absorbing at longer wavelengths for diapause regulation, probably opsin-based—a possibility predicted by Pittendrigh and Minis nearly six decades ago (see quotation from these authors in 1964).

Molecular details of the photoperiodic clock in *N. vitripennis* are quite different to that for *S. argyrostoma*. As with other Hymenoptera, the circadian clock in the wasp is greatly reduced, having lost both *tim* and the photoreceptive form of CRYd during evolution. However, Mukai and Goto (2016) showed that *per* was essential for this species, *per* RNAi females laying eggs that developed into nondiapause larvae even under short days—although female wasps maintained at low temperature did produce diapause destined progeny, suggesting that low-temperature induction differed from that induced by photoperiod. As for photoreception, early action spectrum studies (Saunders 1975b) showed identical responses to monochromatic light, at both early and late phases of the night, with a broad response up to and including that to red light at 617 nm, a situation in contrast to the flesh fly. These data support the contention that the photoperiodic clock of *N. vitripennis* is characteristic of internal coincidence, possibly involving photoreception by opsins.

### Postscript

It is nearly seven decades, since I saw Fig. 3 (p. 15) in Lees’s (1955) Cambridge Monograph *The Physiology of Diapause in Arthropods* showing photoperiodic response curves for various insects. How did they do it? It seemed like a good topic for research. Photoreception and the regulation of diapause became fairly well known during subsequent years, but there was still a gap in the middle—the crux of the problem—or the nature of the time measurement between photoreception and the downstream hormones. Pioneers in the field of photoperiodism, Erwin Bünning (1936) and his enthusiastic supporter, Colin Pittendrigh in the 1950s and 1960s, developed the idea that photoperiodic time measurement was one of the functions of the circadian system. However, results of ‘black box’ experiments were not genes and molecules and some scientists considered that ‘biological rhythms’ were tantamount to something approaching the occult! All that began to change when Konopka (Konopka and Benzer 1971) isolated the *period* mutants of *Drosophila melanogaster*, showing that rhythmicity had a genetic basis, even a single gene having profound effects on the rhythm’s free-running period. The anticipated benefits for diapause research were not immediately forthcoming, however, mainly because *D. melanogaster* had, at best, only a very weak photoperiodic response, more akin to mere quiescence (Saunders et al. 1989). Further progress had to wait several more decades until a more complete repertoire of clock genes in *Drosophila* was characterised, and the similarities (and differences) to other insects possessing robust photoperiodic responses were available. Evidence in favour of two models for the insect photoperiodic clock, internal and external coincidence, then became more apparent. The strength of the external coincidence model, particularly in ‘higher’ Diptera, now offers a good opportunity to elucidate the genetic and molecular aspects of photoperiodic time measurement.
